# Early detection of gastric cancer using global, genome-wide and *IRF4, ELMO1, CLIP4* and *MSC* DNA methylation in endoscopic biopsies

**DOI:** 10.18632/oncotarget.16258

**Published:** 2017-03-16

**Authors:** Francesca Pirini, Sassan Noazin, Martha H. Jahuira-Arias, Sebastian Rodriguez-Torres, Leah Friess, Christina Michailidi, Jaime Cok, Juan Combe, Gloria Vargas, William Prado, Ethan Soudry, Jimena Pérez, Tikki Yudin, Andrea Mancinelli, Helen Unger, Carmen Ili-Gangas, Priscilla Brebi-Mieville, Douglas E. Berg, Masamichi Hayashi, David Sidransky, Robert H. Gilman, Rafael Guerrero-Preston

**Affiliations:** ^1^ Biosciences Laboratory, Istituto Scientifico Romagnolo per lo Studio e la Cura dei Tumori (IRST) IRCCS, Meldola, Italy; ^2^ The Johns Hopkins University, Bloomberg School of Public Health, Department of International Health, Baltimore, MD, USA; ^3^ The Johns Hopkins University, School of Medicine, Otolaryngology Department, Head and Neck Cancer Research Division, Baltimore, MD, USA; ^4^ Universidad Peruana Cayetano Heredia, Lima, Perú; ^5^ Hospital Nacional Cayetano Heredia, Pathology Department, Lima, Perú; ^6^ Instituto Nacional de Enfermedades Neoplásicas, Gastroenterology Department, Lima, Perú; ^7^ Hospital Nacional Arzobispo Loayza, Gastroenterology Department, Lima, Perú; ^8^ Hospital Nacional Dos de Mayo, Gastroenterology Department, Lima, Perú; ^9^ Laboratory of Molecular Pathology, Department of Pathological Anatomy, School of Medicine, Universidad de La Frontera, Temuco, Chile; ^10^ Center of Excellence in Translational Medicine - Scientific and Technological Bioresource Nucleus (CEMT-BIOREN), Universidad de La Frontera, Temuco, Chile; ^11^ Washington University Medical School, Department of Molecular Microbiology, St Louis, MO, USA; ^12^ University of California San Diego, Department of Medicine, La Jolla, CA, USA; ^13^ Department of Gastroenterological Surgery, Nagoya University Graduate School of Medicine, Nagoya, Japan; ^14^ University of Puerto Rico School of Medicine, Department of Obstetrics and Gynecology, San Juan, Puerto Rico

**Keywords:** translational epigenomics, global DNA methylation index, epigenome-wide DNA methylation analysis, IRF4, ELMO1

## Abstract

Clinically useful molecular tools to triage gastric cancer patients are not currently available. We aimed to develop a molecular tool to predict gastric cancer risk in endoscopy-driven biopsies obtained from high-risk gastric cancer clinics in low resource settings.

We discovered and validated a DNA methylation biomarker panel in endoscopic samples obtained from 362 patients seen between 2004 and 2009 in three high-risk gastric cancer clinics in Lima, Perú, and validated it in 306 samples from the Cancer Genome Atlas project (“TCGA”). Global, epigenome wide and gene-specific DNA methylation analyses were used in a Phase I Biomarker Development Trial to identify a continuous biomarker panel that combines a Global DNA Methylation Index (GDMI) and promoter DNA methylation levels of *IRF4, ELMO1, CLIP4* and *MSC*.

We observed an inverse association between the GDMI and histological progression to gastric cancer, when comparing gastritis patients without metaplasia (mean = 5.74, 95% CI, 4.97−6.50), gastritis patients with metaplasia (mean = 4.81, 95% CI, 3.77−5.84), and gastric cancer cases (mean = 3.38, 95% CI, 2.82−3.94), respectively (*p* < 0.0001). Promoter methylation of *IRF4* (*p* < 0.0001), *ELMO*1 (*p* < 0.0001), *CLIP4* (*p* < 0.0001), and *MSC* (*p* < 0.0001), is also associated with increasing severity from gastritis with no metaplasia to gastritis with metaplasia and gastric cancer.

Our findings suggest that *IRF4, ELMO1, CLIP4* and *MSC* promoter methylation coupled with a GDMI>4 are useful molecular tools for gastric cancer risk stratification in endoscopic biopsies.

## BACKGROUND

Gastric cancer (GC) is the fifth most common cancer in both sexes and the third cause of cancer-related death around the world [1]. Chronic infection of the stomach by the bacterium *Helicobacter pylori* leading to chronic inflammation is a major attributable risk factor [2], although less than 2% of *H. pylori* carriers develop gastric cancer [3]. The prognosis of GC is closely related to the stage of disease at the time of diagnosis [4, 5]. The most widely accepted histopathology-based progression model for the development of intestinal-type gastric adenocarcinoma consists of a transition from superficial gastritis to metaplasia to dysplasia and finally, gastric adenocarcinoma [6, 7]. When detected at an early stage, GC is often curable and the five year survival rate is greater than 90%, whereas the prognosis for advanced GC is still poor [8]. Early GC is defined as cancer confined to the mucosa or submucosa regardless of the presence of lymph node metastasis [9], but due to the non specific symptomatology and to the difficulty in distinguishing early GC from benign peptic ulcer or gastritis in the ambulatory setting, less than 20% of GCs are diagnosed at an early stage worldwide [10].

Endoscopy is widely used for the early diagnosis of gastric cancer because of the high accuracy [11], but an accurate reading depends on the endoscopist's observation skills. Apart from conventional endoscopy, magnifying endoscopy combined with narrow-band imaging (NBI) has been recently introduced in the diagnosis of early GC [4, 12] improving the specificity, the sensitivity and the accuracy of the diagnosis [13, 14]. However, missed diagnosis of GC on endoscopy is a common occurrence, with false-negative rates ranging among 5–19% [4, 15, 16]. Consequently, in addition to technical improvements, the identification of novel biomarkers for early diagnosis is urgently needed.

Promoter hypermethylation of several tumor suppressor genes has been correlated with gastric cancer susceptibility [17]. The literature reports many genes significantly hypermethylated in cancer tissue compared with normal tissue of GC subjects [18–20], but relatively few studies have reported DNA methylation markers for early detection [21–24]. Furthermore, quantification of global 5-methylcytosine content [25] and evaluation of long interspersed nuclear elements (LINEs) by Lee [26] and others have emphasized the importance of global DNA hypomethylation in GCs, although little is known about its role in GC.

*H. pylori* infection has been correlated with the progressive accumulation of epigenetic alterations in gastric mucosa [17]. Promoter hypermethylation in specific tumor suppressor genes has been associated with *H. pylori* infection in the multistep carcinogenetic process by several groups [27, 28]. Global DNA hypomethylation also increases throughout the process that leads to the deterioration of normal gastric mucosa to invasive cancer [29–31].

In the present study we used global, genome wide and gene-specific DNA methylation analyses to perform a Phase I Biomarker Development study [32] in order to examine whether gene specific promoter methylation biomarkers, together with a global DNA methylation index (GDMI), could distinguish gastric cancer cases from controls in endoscopic biopsies.

## RESULTS

### Participant characteristics

A total of 376 patients from Peru, mean age 61.2 ± 14.9 years and age range 18–88 years, met the eligibility criteria and were included in our study (Supplementary Tables 1, 2). Patients came to the clinic in Peru with the following symptoms: heartburn (81.8%), belching (76.4%), distension (74.2%), abdominal pain (51.1%), nausea (39.6%), and acid regurgitation (26.3%). There are no significant differences between age and sex in our randomly created Discovery and Validation patient groups: Discovery (Global DNA methylation *n* = 80; Gene-specific DNA methylation *n* = 116) and Validation (Global and Gene-specific DNA methylation *n* = 180) (Supplementary Figure 1A). We performed epigenome-wide DNA methylation analysis of 30 patients selected from the Discovery cohort and Validated the epigenome-wide DNA methylation results from Peru using data from 316 participants in the Cancer Genome Atlas project (Supplementary Figure 1B).

### Biomarker development workflow

Global DNA methylation assays were used to develop the Global DNA Methylation Index (GDMI). The epigenome-wide arrays were used to identify gene-specific promoter regions differentially methylated in cancer, that can be quantified with methylation specific PCR (Supplementary Figure 1C).

### Global DNA methylation and gastric cancer

**Figure 1:**
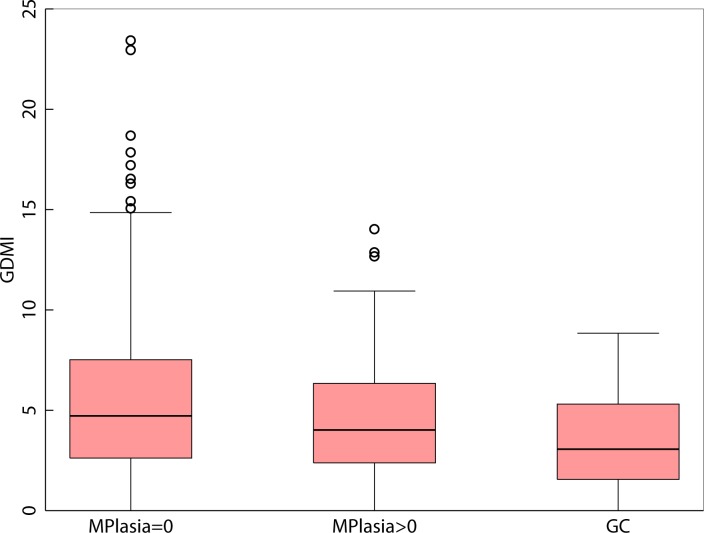
Boxplot of Global DNA Methylation Index levels in gastritis patients without metaplasia, gastritis patients with metaplasia and in patients diagnosed with adenocarcinoma of the stomach

We observed an inverse association (non-parametric trend test for ordered groups *p* < 0.0001) between global DNA methylation in gastritis patients without metaplasia (mean = 5.74), gastritis patients with metaplasia (mean = 4.80), and gastric cancer cases (mean = 3.38) respectively. (*F*-test *p* < 0.0001, Scheffe test *p* < 0.0001, when comparing gastritis patients without metaplasia against GC cases) (Figure 1). In order to assess the GDMI in high-risk GC clinics, we identified global methylation > = 6.5 to be associated with gastritis in a random subset of patients that constituted our discovery set (*n* = 80). Using this criterion for identifying gastric cancer cases in our validation set (*n* = 180) resulted in 84.78% sensitivity and 32.82% specificity with 86.00% negative predictive value (NPV) and 30.71% positive predictive value (PPV) (*p* = 0.023). The same cutoff value for GDMI identified metaplasia > = 10 percent with 87.50% sensitivity, 35.09% specificity, 95.24% NPV and 15.91% PPV (*p* = 0.089). (Supplementary Table 3) When considering the entire data including discovery and validation sets, we identified global methylation > = 9 as the best cutoff point with 100% sensitivity and 100% NPV.

### Epigenome-wide DNA methylation analyses using bump hunting in samples from Peru

#### Gastritis (*n* = 20) vs gastric adenocarcinoma (*n* = 10)

We used the bump hunting method to perform an epigenome-wide analysis of the gastric cancer methylome and identify differentially methylated regions (DMRs) of biological interest using methylation arrays [33, 34]. For epigenome-wide DNA methylation array studies with small sample size (*n* = 3 in each group), bump hunting is the only recommended method for analysis when DNA methylation levels are correlated across CpG loci, as they are in cancer [35]. We found 500 statistically significant DMRs (FWER *p* < 0.05) when we performed epigenome-wide DNA methylation analyses comparing gastritis (*n* = 20) and cancer (*n* = 10) from Perú. Most of the DMRs were observed in chromosome 6 (10%) followed by chromosome 1 (9%), chromosome 19 (8%), chromosome 2 (7%), chromosome 7 (6%), and chromosome 5 (6%). None of the other chromosomes had more than 5% DMRs. We found the DMRs across the following regions of the genome: inside the gene – 431 DMRs (43%); promoter region - 178 DMRs (18%); overlaps 5′ region – 165 DMRs (16%); downstream from TSS – 114 DMRs (11%); upstream from TSS – 111 DMRs (11%). The number of individual CpGs per DMR ranged from 1 to 18. Most of the DMRs had fewer than 10 CpGs (97%). The DMRs with more than 10 CpGs were found overlapping the downstream region of the first exon (16 DMRs), inside the exon (5 DMRs), covering the exon (3 DMRs), overlapping the upstream region of the first exon (1 DMR), inside the intron (1 DMR), and overlapping two exons (1 DMR).

### Epigenome-wide DNA methylation analyses using bump hunting in samples from TCGA

We found 695 statistically significant DMRs (FWER *p* < 0.05) when we performed epigenome-wide DNA methylation analyses comparing normal (*n* = 11) and cancer (*n* = 295) samples from TCGA. Most of the DMRs were observed in chromosome 1 (12%) followed by chromosome 6 (9%) and chromosome 2 (7%). We found the DMRs across the following regions of the genome: inside the gene – 135 DMRs (53%); promoter region - 58 DMRs (23%); overlaps 5′ region – 30 DMRs (12%); downstream from TSS – 19 DMRs (7%); upstream from TSS – 11 DMRs (4%). The number of individual CpGs per DMR ranged from 1 to 10. Most of the DMRs had fewer than 4 CpGs (94%). The DMRs with more than 4 CpGs were found overlapping the downstream region of the first exon (8 DMRs), inside the exon (6 DMRs), inside the intron (3 DMRs), and covering the exon (1 DMR).

### Gene-specific DNA methylation in DMRs associated with gastric cancer in Peru and TCGA datasets

**Figure 2:**
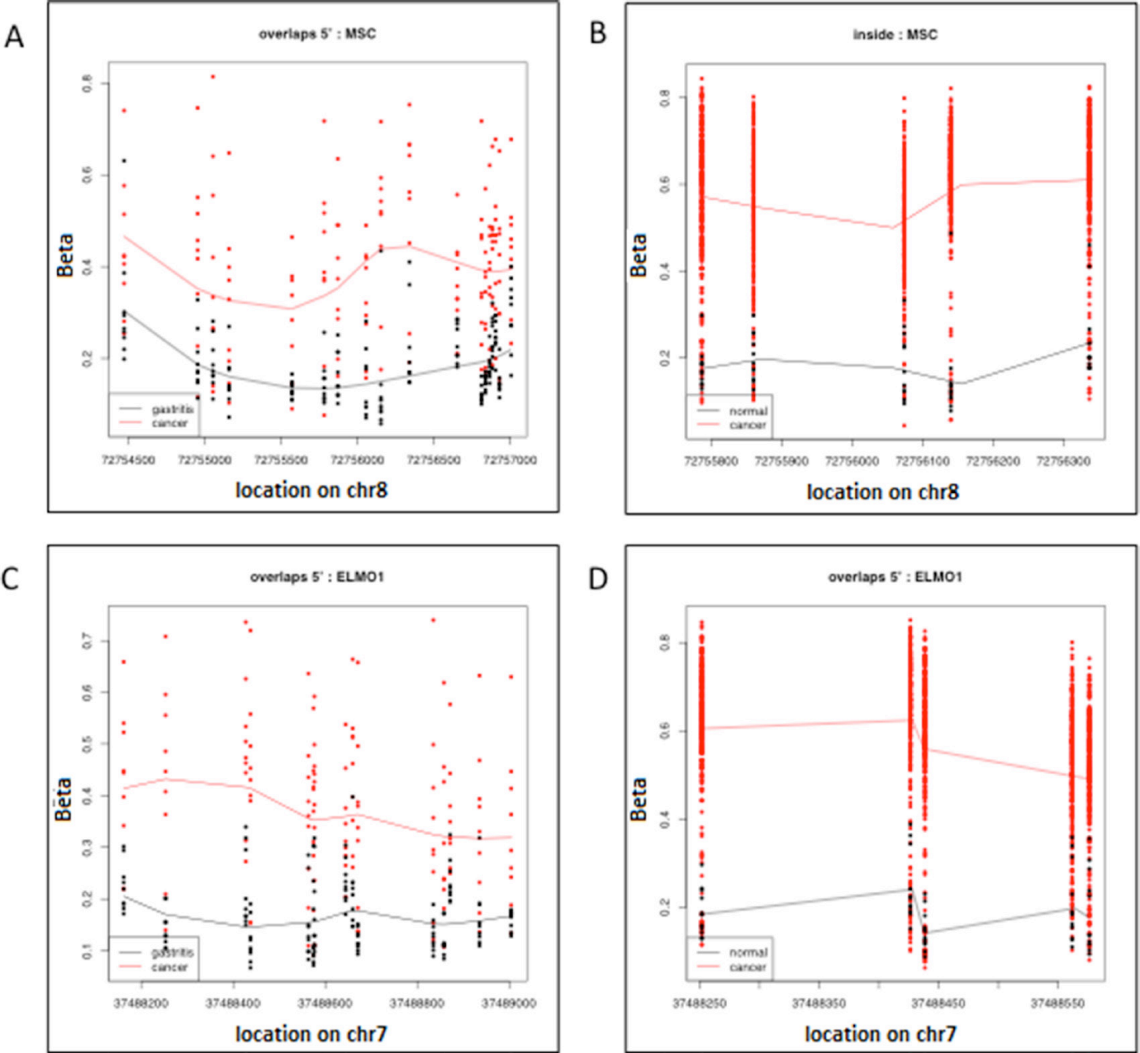
Significant Differentially Methylated Regions (DMR) identified with the bump hunting algorithm, as implemented in the minfi package from Bioconductor Each row represents an individual CpG and each data point represents an individual patient. (**A**) Shows the DMR located on chromosome 8, which has the approximate genomic coordinates starting (72754500) and end (7275700) points. The genomic coordinates of this DMR correspond to the 5′ end of the Musculin (MSC) gene in the hg19 human genome build. This DMR was found to be differentially methylated when comparing the genome-wide epigenome of 10 gastric adenocarcinoma samples (red) and 20 gastritis samples (black) from patients seen in a high-risk gastric cancer clinic in Peru; (**B**) shows the differentially methylated CpGs (each row) located on chromosome 8 with approximate starting (72755800) and end (72756300) genomic coordinates. The genomic coordinates of this DMR correspond to the 5′ end of the Musculin (MSC) gene and are located within the DMR in 1A. This DMR was found to be differentially methylated when comparing the genome-wide epigenome of 295 gastric adenocarcinoma samples (red) and 11 gastritis samples (black) from patients participating in the Cancer Genome Atlas (TCGA) project; (**C**) shows the DMR located on chromosome 8 with approximate starting (37488200) and end (37489000) genomic coordinates. The genomic coordinates of this DMR correspond to the 5′ end of the Engulfment and Cell motility 1 (ELMO1) gene. This DMR was found to be differentially methylated when comparing the genome-wide epigenome of 10 gastric adenocarcinoma samples (red) and 20 gastritis samples (black) from patients seen in a high-risk gastric cancer clinic in Peru; (**D**) shows the differentially methylated CpGs (each row) located on chromosome 8 with approximate starting (37488250) and end (37488550) genomic coordinates. The genomic coordinates of this DMR correspond to the 5′ end of the Engulfment and Cell motility 1 (ELMO1) gene and are located within the DMR in (A). This DMR was found to be differentially methylated when comparing the genome-wide epigenome of 295 gastric adenocarcinoma samples (red) and 11 gastritis samples (black) from patients participating in the Cancer Genome Atlas (TCGA) project.

We used qMSP to confirm promoter methylation in four of the 390 statistically significant DMRs (FWER *p* < 0.05) common to both the Peru and TCGA sample sets: *IRF4, ELMO1, CLIP4* and *MSC*. Interestingly, there is very little difference in the genomic coordinates seen in the X-axis of the scatterplots that draw the significant *ELMO1* and *MSC* DMRs identified by two separate epigenome-wide analyses of the samples from Peru and TCGA, performed with the bump hunting algorithm in minfi (Figure 2). This overlap in genomic coordinates for DMRs identified by separate epigenome-wide analyses of samples from patients with different ethnic, racial and socio-economic characteristics, suggest that promoter methylation of *ELMO1* and *MSC*, may track biological changes associated with adenocarcinoma, regardless of life-style and environmental exposures.

**Figure 3:**
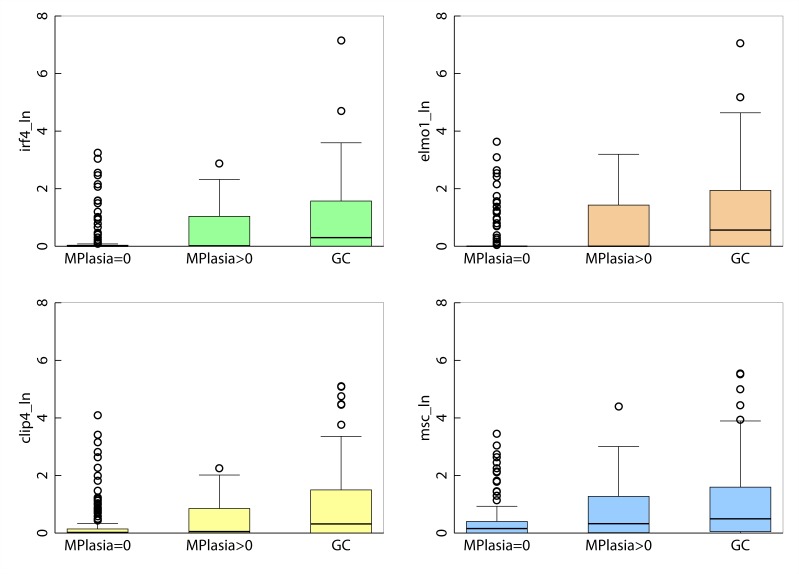
Boxplot of gene-specific promoter methylation in gastritis patients without metaplasia, gastritis patients with metaplasia and in patients diagnosed with adenocarcinoma of the stomach

Natural log values of promoter methylation in *IRF4, ELMO1, CLIP4* and *MSC* quantified with qMSP, increase with age but are not gender dependent (Supplementary Figure 2). Additionally, promoter methylation levels for all four genes are highly correlated among themselves, suggesting the possibility they share a common epigenetic clustering factor in their clonal evolution (Supplementary Table 4). Promoter methylation of *IRF4* (*p* < 0.001)*, ELMO1* (*p* < 0.001)*, CLIP4* (*p* < 0.001), and *MSC* (*p* < 0.001), is strongly associated with increasing severity of disease in the histological progression from gastritis with no metaplasia, to gastritis with metaplasia, to gastric adenocarcinoma (Figure 3). Using logistic regression, we observed a statistically significant association between gastritis patients with metaplasia > = 10% and promoter methylation of *IRF4, ELMO1* and *MSC*, after adjusting for age and sex. We also observed a statistically significant association between gastric cancer diagnosis and promoter methylation of *IRF4, ELMO1, CLIP4* and *MSC*, after adjusting for age and sex (Table 1). Together these results suggest that promoter methylation of *IRF4, ELMO1, CLIP4* and *MSC* may be part of a gastric cancer CpG island methylator phenotype (CIMP). The concept of CIMP)was introduced to refer to a subset of colorectal carcinomas (CRCs) with widespread methylation of numerous promoter CpG island loci. Akin to microsatellite instability (MSI), CIMP is now recognized as one of the most important molecular carcinogenesis pathways of CRCs, and CIMP-high (CIMP-H) CRCs have been characterized for their clinicopathological features [36].

**Table 1:** Promoter DNA methylation association with gastric cancer and metaplasia > = 10%

	Odds Ratio	Sth. Err.	95% Conf. interval	Pseudo R2	Obs	*P*
**Gastric cancer (unadjusted)**						
gmeth_ln	0.49	0.10	0.32–0.73	0.04	257	< 0.0001
*IRF4*_ln	2.37	0.38	1.72–3.25	0.10	291	< 0.0001
*ELMO1*_ln	2.00	0.27	1.54–2.59	0.06	286	< 0.0001
*CLIP4*_ln	1.90	0.27	1.43–2.51	0.07	290	< 0.0001
*MSC*_ln	1.76	0.24	1.35–2.30	0.06	291	< 0.0001
**Gastric cancer (age, sex adj)**						
gmeth_ln	0.46	0.10	0.30–0.71	0.09	249	< 0.0001
*IRF4*_ln	2.21	0.37	1.60–3.07	0.12	276	< 0.0001
*ELMO1*_ln	1.95	0.27	1.48–2.57	0.12	271	< 0.0001
*CLIP4*_ln	1.78	0.26	1.33–2.38	0.09	275	< 0.0001
*MSC*_ln	1.67	0.24	1.26–2.21	0.09	276	< 0.0001
**Mplasia > = 10% (unadj)**						
gmeth_ln	0.50	0.15	0.29–0.89	0.04	183	0.018
*IRF4*_ln	2.49	0.68	1.46–4.25	0.08	188	0.001
*ELMO1*_ln	2.44	0.55	1.57–3.79	0.12	183	< 0.0001
*CLIP4*_ln	2.08	0.53	1.27–3.41	0.06	187	0.004
*MSC*_ln	2.61	0.64	1.61–4.24	0.12	188	< 0.0001
**Mplasia > = 10% (age, sex adj)**						
gmeth_ln	0.43	0.13	0.23–0.79	0.09	176	0.007
*IRF4*_ln	1.89	0.58	1.03–3.44	0.11	177	0.038
*ELMO1*_ln	1.88	0.47	1.15–3.08	0.13	172	0.012
*CLIP4*_ln	1.57	0.43	0.92–2.69	0.10	176	0.098
*MSC*_ln	1.98	0.54	1.17–3.36	0.13	177	0.011

### Predictive models with gene-specific methylation

We used the gene specific methylation association with gastric cancer within the discovery dataset (*n* = 116) to estimate separate screening models using *IRF4, ELMO1, CLIP4* or *MSC* to classify participants in the validation set who were identified as having a high risk of gastric cancer based on a GDMI < 6.5. This resulted in 74% correct classification as indicated in Table 2.

**Table 2:** Odds ratios and coefficient of models predicting GC risk using IRF4, ELMO1, CLIP4 or MSC

	OR	SE	95% Conf int		Coef	SE	95% Conf int		*P*
**IRF4**	1.398	0.236	1.003	1.947	0.335	0.169	0.003	0.667	0.048
**const**					−0.317	0.208	−0.723	0.09	0.127
**ELMO1**	1.195	0.104	1.007	1.418	0.178	0.087	0.007	0.349	0.041
**const**					−0.293	0.211	−0.706	0.121	0.166
**CLIP4**	1.136	0.091	0.971	1.328	0.127	0.08	−0.029	0.284	0.111
**const**					−0.225	0.203	−0.624	0.174	0.269
**MSC**	1.249	0.155	0.979	1.594	0.223	0.124	−0.208	0.466	0.073
**const**					−0.334	0.216	−0.756	0.09	0.123

### Subset analysis of patients misdiagnosed by endoscopists

Endoscopists diagnosed a total of 201 patients with high-risk gastric cancer. More than half of the patients (53%) were diagnosed as gastric cancer patients (*n* = 80) by endoscopists. Pathologists at two separate institutions subsequently revised the diagnosis in 30 of the 80 cancer patients (37.5%) diagnosed by the endoscopists in high-risk clinics, as gastritis patients, using the updated Sydney system for the histologic classification of gastritis. Thus, subset analysis was performed on 50 gastric adenocarcinoma patients in the case study group and 151 gastritis patients in the control study group.

**Figure 4:**
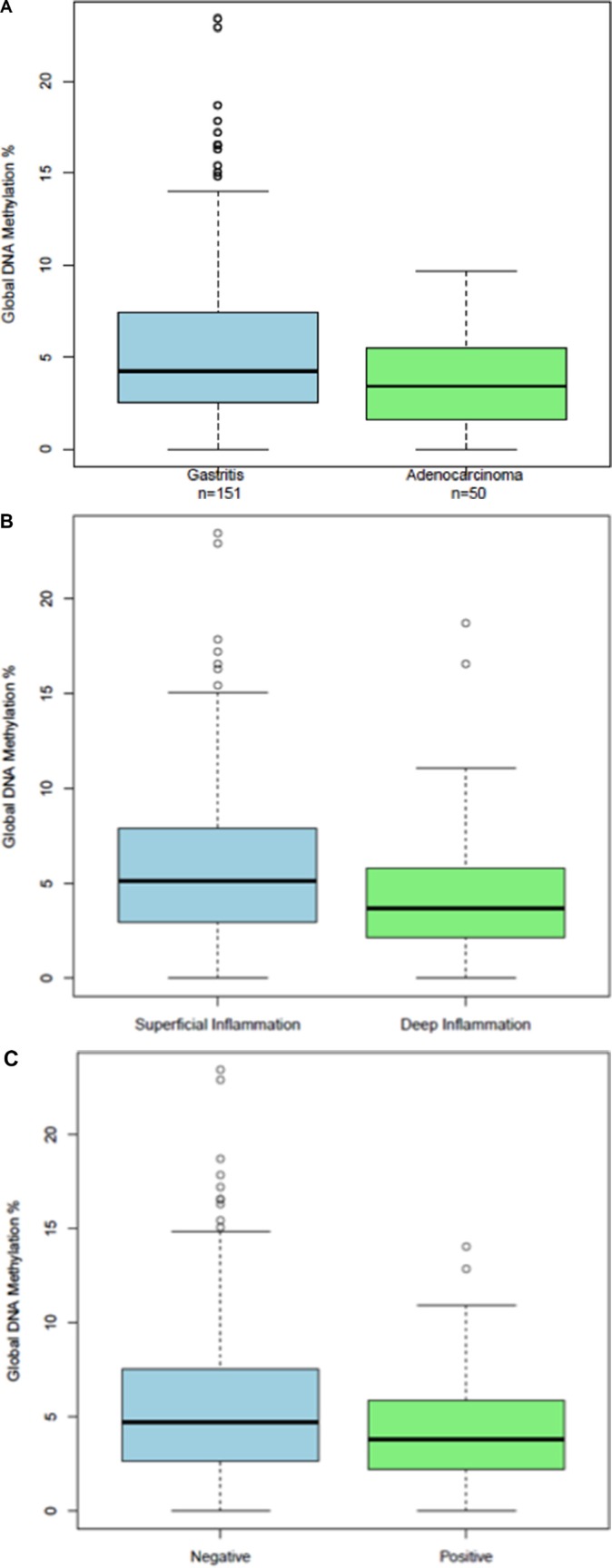
(**A**) Boxplots of Global DNA Methylation Index levels in a subset of adenocarcinoma cases and gastritis controls that were diagnosed by endoscopists in the clinic; (**B**) Boxplots of Global DNA Methylation Index levels in a subset of gastritis patients dichotomized according to the depth of superficial and deep inflammation; (**C**) Boxplots of Global DNA Methylation Index levels in deep inflammation cases according to the presence of intestinal metaplasia.

We found that the GDMI discriminates between diagnosis of gastric cancer and gastritis by endoscopists. As can be seen in Figure 4A, global DNA methylation levels were significantly lower in gastric cancer cases than in gastritis controls (*p* = 0.002). The mean GDMI for the controls was 5.7 (95% CI, 4.93–6.41) and the mean GDMI for the cases was 3.7 (95% CI, 2.99–4.39). The summary statistics for this comparison are listed in the Supplementary Table 5.

The pathological characteristics of the gastritis patients comprising the control study group are summarized in Supplementary Table 6. The revised Sydney system for classification of gastritis patients was used to distinguish between gastritis patients with superficial and deep Inflammation. Briefly, in gastritis patients with superficial inflammation the chronic active infiltration occupies the lamina propria between the gland crypts but remains above the glands necks. The glands do not show alterations. In gastritis patients with deep inflammation the chronic active infiltration extends beyond the gastric gland neck, occupying the space between the glands. The glands themselves do not show alterations. Gastritis patients with deep inflammation had a larger percentage of moderate and severe inflammation, a much higher frequency of atrophy and intestinal metaplasia, and a much lower incidence of *helicobacter pylori* (Supplementary Table 6).

We found that deep inflammation is associated with global DNA hypomethylation, a hallmark of human cancer: two-sided *p* = .097, one-sided *p* = .0467. (Figure 4B). The GDMI was able to discriminate gastritis patients according to the depth of inflammation: *t*-test (*p* = 0.01) and Mann-Whitney (*p* = 0.02). The mean GDMI for gastritis patients with superficial inflammation was 6.42 (95% CI, 5.3–7.5) compared to 4.65 (95% CI, 3.74–5.57) for those with deep inflammation. Furthermore, among gastritis patients with deep inflammation we found a significant association (*p* = 0.03) in the GDMI when comparing patients with and without intestinal metaplasia (Figure 4C). Gastritis patients with deep inflammation and intestinal metaplasia had significantly (*p* = 0.03) lower global DNA methylation levels (mean = 3.7, 95% CI, 2.75–4.73) when compared to those without intestinal metaplasia (mean = 5.5, 95% CI, 3.98–6.93).

### Autosomal epigenome-wide analysis - misdiagnosed gastritis (*n* = 10) vs gastric adenocarcinoma (*n* = 10)

We found 1448 statistically significant DMRs (FWER *p* < 0.05) when we performed epigenome-wide DNA methylation analyses comparing gastritis samples misdiagnosed as gastric adenocarcinoma by endoscopists (*n* = 10) and gastric adenocarcinoma (*n* = 10) from Perú. Most of the DMRs were observed in the X chromosome (36%) followed by chromosome 6 (6%) and the Y chromosome (6%). None of the other chromosomes had more than 5% DMRs. We found the DMRs across the following regions of the genome: inside the gene – 639 DMRs (44%); overlaps 5′ region - 295 DMRs (20%); promoter region – 260 DMRs (18%); downstream from TSS – 133 DMRs (9%); upstream from TSS – 118 DMRs (8%). The number of individual CpGs per DMR ranged from 1 to 18. Most of the DMRs had fewer than 10 CpGs (96%). The DMRs with more than 10 CpGs were found overlapping the downstream region of the first exon (39 DMRs), inside the exon (10 DMRs), and covering the first exon (5 DMRs).

### Autosomal epigenome-wide-misdiagnosed gastritis (*n* = 10) gastritis (*n* = 10)

We found 392 statistically significant DMRs (FWER *p* < 0.05) when we performed epigenome-wide DNA methylation analyses comparing gastritis (*n* = 10) and gastritis samples misdiagnosed as gastric adenocarcinoma by endoscopists (*n* = 10) from Perú. Most of the DMRs were observed in the X chromosome (27%) followed by chromosome 6 (10%) and chromosome 1 (6%). None of the other chromosomes had more than 5% DMRs. We found the DMRs across the following regions of the genome: inside the gene – 428 DMRs (46%); promoter region - 180 DMRs (19%); overlaps 5′ region – 139 DMRs (15%); upstream from TSS – 93 DMRs (10%); downstream from TSS – 82 DMRs (9%). The number of individual CpGs per DMR ranged from 1 to 14. Most of the DMRs had fewer than 7 CpGs (96%). The DMRs with more than CpGs were found overlapping the downstream region of the first exon (15 DMRs) and inside the first exon (11 DMRs).

## DISCUSSION

**Figure 5:**
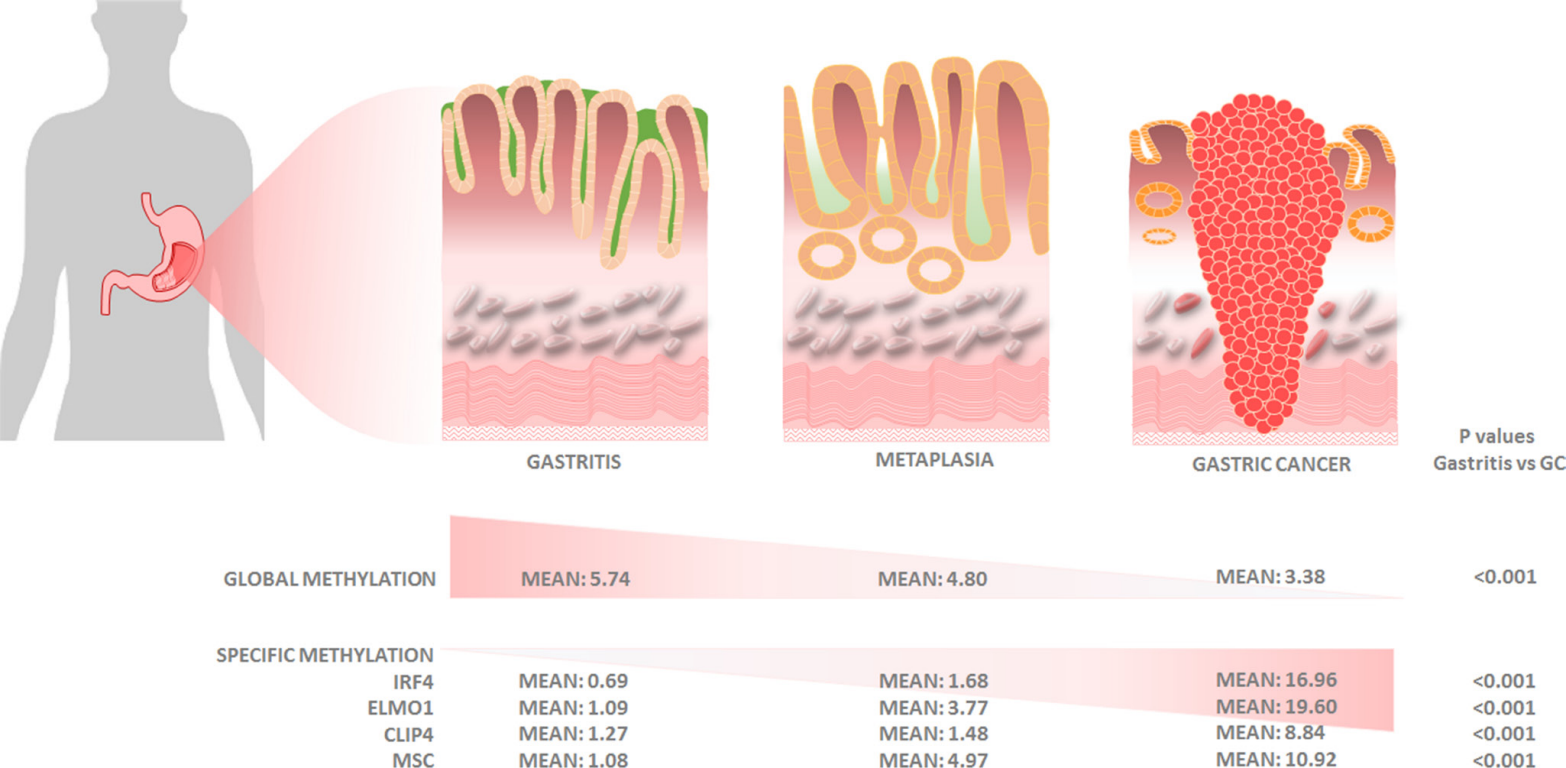
Graphical representation of histologic and molecular progression stages proposed for gastric adenocarcinoma, with mean values for global and gene-specific promoter methylation of *IRF4, ELMO1, CLIP4* and *MSC*

Ours is the first study that combines global, epigenome-wide and gene-specific methylation analyses of the gastric cancer epigenome in endoscopic biopsies obtained from ambulatory high-risk gastric cancer clinics. Our findings suggest that promoter methylation of *CLIP4, IRF4, ELMO1*, and *MSC*, together with a GDMI > 4, is a useful molecular panel for gastric cancer risk stratification in endoscopic biopsies (Figure 5).

Promoter methylation of CAP-Gly Domain Containing Linker Protein Family Member 4 (*CLIP4*) is the only one, of the four genes in our panel, which has been reported as methylated in gastric cancer in previous studies. *CLIP4*, also known as *UBASH3A* or *TULA*, is a member of the T-cell ubiquitin ligand family and suppresses T-cell signaling. *CLIP4* can facilitate growth factor withdrawal-induced apoptosis in T-cells [37] and promote the accumulation of various activated target receptors, such as T-cell receptors, epidermal growth factor (EGF) receptor, and platelet-derived growth factor beta-receptor [38, 39], which can induce cell invasiveness and metastasis. *CLIP4* also activates the EGFR signaling pathway by downregulation of the EGF receptor [40]. In other cells, *CLIP4* activates *Syk* [41], a member of the protein tyrosine kinase family linked with cell motility and increased cell migration [42–44].

Interferon regulatory factors (IRFs) are a family of transcriptional regulators defined by a characteristic homology in their DNA-binding domain. They play an important role in the regulation of various genes (such as IFNs, interleukins, MHC class I/II), apoptosis and differentiation/maturation [45, 46]. Interferon regulatory factor 4 (*IRF-4*) is one member with very restricted expression pattern and plays a crucial role in the function of immune cells. Predominately B- and activated T-lymphocytes are *IRF-4* positive [47]. Deletion of *IRF-4* causes failure to develop mature and functionally active B- and T-lymphocytes [48].

Engulfment and cell motility 1 (*ELMO1*) plays a role in promoting cancer cell migration and invasion in malignant glioma [49]. *ELMO1* promoter methylation roles are also reported in human colorectal cancer [50], kidney disease [51], and rheumatoid arthritis [52]. The protein produced by *ELMO1* belongs to a protein family that interacts with dedicator of cytokinesis proteins to promote phagocytosis and cell migration.

Musculin, a human protein encoded by the *MSC* gene, is a transcriptional repressor capable of binding an E-box element either as a homodimer or as a heterodimer with E2A *in vitro*. The encoded protein also forms heterodimers with E2A proteins *in vivo*. This protein is capable of inhibiting the transactivation capability of E47, an E2A protein, in mammalian cells. This gene is a downstream target of the B-cell receptor signal transduction pathway [53, 54]. MSC (and probably TCF21) is located together with TBX1 and PITX2 upstream of myogenic regulatory factor 5 (*Myf5*) and Myogenic Differentiation 1 (*MyoD 1*). MSC regulates the levels of expression of *Myf5* and *MyoD* by direct interactions through ubiquitin-activating enzymes E1 C-terminal related (*ECR-1*) and the distal regulatory region (DRR), respectively [55]. Methylation of Musculin has not been previously reported.

Methylation of individual dinucleotide cytosine-guanosine motifs (CpG) in CpG islands located in promoter regions, is one of the mechanisms of gene regulation in mammals and a common event of gene silencing in cancer. Gain of methylation of CpG islands together with global loss of methylation are frequently observed events in every tumor cell, in contrast to normal cells [56, 57]. *De novo* DNA methylation of genes with important cellular regulatory functions, such as cell cycle, DNA repair, apoptosis and tumor suppressor genes, is involved in tumorigenesis [58–60].

Several gene specific DNA methylation events seen in gastritis and gastric cancer patients may be associated with *Helicobacter pylori* infection and the progression of dysplastic lesions to adenocarcinoma of the stomach, but the evidence is contradictory and non-conclusive yet. Promoter methylation in a subset of gastric cancer patients is associated with the CpG island methylator phenotype, which includes methylation of such genes as *CDKN2A* and *hMLH1* [61, 62]. Promoter methylation in *Helicobacter pylori* or *Epstein-Barr* virus infected gastric mucosae may play a functional role and lead to gastric cancer risk [27, 63–65]. Inactivation of *COX-2, HMLH1* and *CDKN2A* by promoter methylation depends on the *Helicobacter pylori* genotype and occurs by distinct pathways, according to the histological subtype and tumor location [66].

Promoter methylation of MGMT is related to gastric cancer risk, distant metastasis, and lymph node metastasis, which indicates that MGMT promoter methylation may play an important role in gastric cancer development [67]. Promoter methylation of ASC/TMS1 is associated with poor prognosis of patients with gastric cancer [68]. *RBL1* is methylated in patients with intestinal metaplasia, with or without *Helicobacter pylori*, and in gastric cancer patients [69]. Promoter methylation of RNF180 is associated with *Helicobacter pylori* infection and serves as a marker for gastric cancer and atrophic gastritis [70].

Promoter methylation of *RNF180, DAPK1* and *SFRP2* can be detected in plasma DNA of patients with gastric cancer [71]. Promoter methylation of *E-cadherin* in a subset of our gastric cancer patients is consistent with aggressiveness and metastasis of gastric cancer [72]. *HLTF* methylation plays a role in the early stages of gastric carcinogenesis in patients with family histories and may be a valuable susceptible marker for the risk of gastric cancer in individuals with family predisposition [73].

Several studies have been carried out examining altered methylation levels in normal gastric mucosa and GC attempting to identify possible biomarkers for the surveillance of high-risk patients. Bernal et al., showed that aberrant hypermethylation of the *Reprimo* gene represents a potential biomarker for the early detection of gastric cancer with differential methylation in the plasma DNA between controls and cases. Moreover, the signet-ring cell-type of GC was associated with a methylation profile of eight specific genes [74]. Also, promoter methylation levels are correlated with loss of expression of *Reprimo* in gastric cancer tissues, progression from stage I to stages II–IV and lymph node metastasis [75].

Other studies, using epigenome-wide DNA methylation arrays for discovery and qMSP for validation, unveiled the role of promoter methylation in in endoscopic samples taken from high-risk clinics patients diagnosed with gastritis and gastric carcinogenesis. Shin et al. reported the promoter methylation of *MOS, DCC, CRK*, and *PTPN6* in gastric cancer [76]. Epigenome-wide DNA methylation arrays have also identified the role of *DVL2* and *ETS1* methylation in diffuse- and mixed-types of early gastric cancers [77]. On the other hand, *C19orf35* and *CNRIP1* were related to the diffuse type rather than intestinal type, and *GAL3ST2* and *ITGA3* were related to the mixed-type rather than the other two types [77]. The methylation of other genes, *CLIP4, XKR6, CCDC57, MAML3* and *SDC2*, was related with one or more of the following variables: age, tumor location, and *Helicobacter pylori* infection, rather than with the histologic subtype [77].

We also investigated the role of GDMI as biomarker for gastric cancer carcinogenesis. The lower GDMI levels we observed in gastric cancer biopsies are consistent with the observations made by Lee et al, namely that gastric epithelial dysplasia and intramucosal cancer tissues had significantly lower levels of LINE-1 methylation than adjacent normal gastric tissues [26]. LINE-1 is a surrogate marker of global DNA methylation that measures methylation in a subset of repetitive elements across the genome. The GDMI is a better indicator of global DNA methylation levels than LINE1, because it quantifies DNA methylation across the entire human genome. Our study further shows that the GDMI can discriminate gastritis patients with different degrees of inflammation and metaplasia. This interesting molecular difference suggests the ability to potentially discriminate between different degrees of gastritis severity, intestinal metaplasia and gastric adenocarcinoma, solely based on global DNA methylation levels in gastric mucosa epithelium.

The GDMI can discriminate the depth of inflammation, as gastritis patients with deep inflammation exhibit lower GDMI than those with superficial inflammation, suggesting that at the molecular levels these patients may be the most prone to progress towards gastric cancer. We also observed interesting results when contrasting patients with intestinal metaplasia less than or greater than 10%. Patients with intestinal metaplasia > = 10% had lower GDMI (*p* = 0.013). We also found that the gastritis patients who were originally diagnosed with cancer by the endoscopists harbor lower GDMI values than the gastritis patients, classified as such by both the endoscopists and the pathologists. Furthermore, these gastritis patients misdiagnosed as cancer patients by endoscopists had a similar global DNA methylation profile as adenocarcinoma patients. These data support the notion that the misdiagnosed cases have an underlying oncogenic molecular process and are most likely to deteriorate to cancer. It should be noted that in all of our models of gastric cancer, sex was a significant covariate while age was not. This does not reflect absence of association between gastric cancer and age, but rather the correlation between age and GDMI, which partially accounts for the covariation between age and the outcome, masking some of the contribution by age. By contrast, sex and GDMI were independent.

Only a few studies have examined global DNA hypomethylation in gastric cancer by itself, under the rationale that hypomethylation is the earliest epigenomic event that signifies the transition from a normal to a malignant phenotype. While the precise mechanisms that lead to a global loss of methylation patterns in cancer are still to be elucidated, it is evident that global DNA hypomethylation is already present in the early stages of gastric carcinogenesis and may play various roles in the biologic progression of gastric cancer lesions. Genome wide hypomethylation and regional hypermethylation have been shown to occur in an enlarged-fold gastritis that may also contribute to the tumorigenesis of diffuse-type gastric cancers [78]. Another study found that global DNA hypomethylation, assessed by incubating DNA with (3H)-S-adenosylmethionine and Sss1 methylase, also occurs in the early stages of gastric carcinogenesis up to chronic atrophic gastritis, but is lost as a marker in gastric cancer. They also found that global hypomethylation of DNA increased substantially with severe atrophy (*p* = 0.01) or with type III intestinal metaplasia (*p* = 0.15), thus leading the authors to propose it as a useful biomarker of gastric neoplasia, and monitoring the response to chemopreventive agents [79]. Another study found that LINE-1 hypomethylation is significantly associated with *H. pylori* infection, supporting a role for LINE-1 methylation level as viable epigenetic marker of gastric mucosae induced by *H. pylori* infection [31]. Collectively, these studies suggest that the GDMI may be an epigenomic reflection of important early cellular events in gastric cancer initiation and progression [80, 7].

Much as with mutational data, our data on global, epigenome-wide and promoter DNA methylation alterations in gastric premalignant lesions, should encourage further analyses of differential methylation in specific genetic loci associated with over and under expression in signaling pathways that contribute to gastric cancer development or progression, and how these loci match or differ from those implicated in other types of cancers, all of which in turn could have implications for development of new early detection and diagnostic tools for the precision medicine era.

## MATERIALS AND METHODS

### Participants

The study population consists of a prospective, observational cohort initiated in 2006 and closed in June 2009. We recruited 576 patients who were seen by gastrointestinal endoscopists in high-risk gastric cancer clinics of the Gastroenterology Divisions at three hospitals in Lima: Hospital Nacional Arzobispo Loayza, Hospital Nacional Dos de Mayo, and the cancer-specialized Instituto Nacional de Enfermedades Neoplásicas. The inclusion criterion for the gastritis controls in the study was for the patients to have gastro-duodenal symptoms (ICD9-CM code 535) whereas for the cases, the inclusion criterion was to have a clinical diagnosis of gastric cancer (ICD9-CM code 151), all determined by pathologists at two different institutions in Peru. A pathologist at Johns Hopkins School of Medicine reviewed a random sample of slides. All patients underwent endoscopic diagnosis and biopsy. From this cohort, 376 study patients had DNA samples adequate for analysis. Participants were randomly assigned to three groups: a Discovery group for GDMI analysis; a Discovery group for epigenome-wide and gene-specific DNA methylation analysis; and a Validation group for combined GDMI and gene-specific promoter DNA methylation analysis. The Institutional Review Boards of the Instituto Nacional de Neoplásicas, the National Hospital Arzobispo Loayza, the Hospital Nacional Dos de Mayo, the Universidad Peruana Cayetano Heredia and the Johns Hopkins School of Medicine (NA_00020633) approved the research protocols. Informed written consent was obtained from all patients included in the study.

### Tissue samples and DNA extraction

Two endoscopy biopsies were taken from each patient. Biopsies were obtained from the cancerous lesion for the cases and from the gastric antrum for the controls, frozen at −70 and sent for processing and storage in the Universidad Peruana Cayetano Heredia. Gastric mucosa tissue was fixed in 10% formalin buffer and embedded in paraffin for microscopy histological examination. Hematoxylin and eosin-stained histological slides were scored using the Sydney System (Lash, 2013 #166). Biopsies indicative of intestinal metaplasia were stained with PAS. *H. pylori* lesions were identified by Warthin Starry silver stain. All neoplastic tissues used in this study were classified as gastric adenocarcinoma by histopathology.

DNA was extracted from the frozen tissue samples using the QIAmp DNA Mini Kit (QIAGEN, Germany) and stored at −20°C until use in the Universidad Peruana Cayetano Heredia. DNA concentrations were measured using Nanodrop ND-1000 spectrophotometer. Five micrograms of DNA were sent to Johns Hopkins University for DNA methylation analyses.

### Global DNA methylation analysis

Global DNA methylation levels were determined by ELISA using the MDQ1, Imprint^®^ Methylated DNA Quantification Kit (Sigma, USA) according to the manufacturer's instructions. Each analysis for sample(s) and control DNA was performed in duplicate and the average of the absorbance readings at 450nm (A_450_) was used for calculations. The GDMI for each sample was calculated according to the equation: [(A_450av_Sample - A_450av_Blank)/(A_450av_Methylated Control DNA - A_450av_Blank)] × 100.

### Epigenome-wide DNA methylation analysis

The HumanMethylation450K DNA BeadChip assay was used to perform unbiased epigenome-wide DNA methylation analysis. Bisulfite modification of genomic DNA (2 μg) was performed with EpiTect Bisulfite Kit (QIAGEN) according to the manufacturer's protocol. We hybridized bisulfite converted DNA to the HumanMethylation450K array to identify differentially methylated regions (DMRs) in gastritis samples (*n* = 10), gastritis samples misdiagnosed as cancer by endoscopists (*n* = 10) and cancer samples (*n* = 10). To validate these results we performed an unbiased epigenome-wide DNA methylation analysis to identify DMRs in gastric cancer samples (*n* = 295) from the Cancer Genome Atlas project (TCGA) and gastritis controls (*n* = 20) from Perú.

We imported the data into R using the illuminaio package [81]. For data normalization we used the minfi package to apply the Noob background subtraction and dye-bias correction [82] followed by normalization and identification of DMRs between cases and controls using the bump hunting method in minfi [83]. The minfi package provides tools for analyzing Illumina's methylation arrays and includes methods for preprocessing, quality assessment, and detection of differentially methylated regions from the kilobase to the megabase scale. We performed pre-processing with the minfi package applying a version of subset quantile normalization to the Meth and Unmeth intensities separately. The distribution of type I and type II probes were forced to be the same by first quantile normalizing the type II probes across samples and then interpolating a reference distribution to which the type I probes are normalized. The stratified quantile normalization method is implemented by the preprocessQuantile function (the function does no background correction and removes zeros using the fix2 MethOutlier function). This algorithm relies on the assumptions necessary for quantile normalization and involves both within and between sample normalization. After normalization the bump hunting method was carried out by first fitting a linear regression model for each locus before smoothing the coefficient within clusters along the genome to identify bumps. More specifically, for each locus, a linear model was used to estimate the coefficient of difference in methylation levels between the cancer group and the normal groups. After fitting the linear regression model, the bump hunting method was implemented to estimate the regions for which there were statistically significant differences in methylation levels (Differentially Methylated Regions or DMRs) between the cancer group and the normal groups. Statistical uncertainty was assigned to each DMR using permutation tests generate a raw *p*-value from the bump hunting method, and an adjusted *p*-value generated from the minfi package using Storey's procedure (bump hunting *q*-value). We then intersected the statistically significant DMRs (FWER *p* < 0.05) that discriminate gastric cancer from gastritis in samples from Peru and TCGA.

### Gene-specific DNA methylation analyses

We selected four of the genes that had the greater variance and the largest number of CpGs in the DMR window, from the list of significant DMRs (FWER *p* < 0.05) common to both the DMR results from the Peru and TCGA epigenome-wide analyses: *IRF4* (interferon regulatory factor 4); *ELMO1* (encodes Engulfment and cell motility protein 1); *CLIP4* (CAP-Gly domain containing linker protein family member 4); and *MSC* (encoding Musculin protein). We designed primers and probes to quantify promoter methylation of these four genes using fluorogenic quantitative methylation specific PCR (qMSP), as previously described [84].

Briefly, bisulfite-modified DNA was used as a template for qMSP. The genomic sequence for the genes within the DMRs identified with the bump hunting algorithm and 1000 bases up- and downstream was obtained from the UCSC Genome Browser [85]. The primers and hybridization probes for methylation analysis were designed based on this sequence by using MethPrimer [86]. All primer and probe sequences are listed in Supplementary Table 8. Fluorogenic PCR reactions were performed in duplicates in a reaction volume of 20 μl that contained 3 μl of bisulfite-modified DNA; 600 nM of each primer; 200 nM probe; 0.75 U of platinum Taq polymerase (Invitrogen, MD, USA); 200 μM of each dATP, dCTP, dGTP and dTTP; 200 nM ROX dye reference; 1X buffer (16.6 mM ammonium sulfate; 67 mM Trizma [Sigma]; 6.7 mM of magnesium chloride; 10 mM of mercaptoethanol and 0.1% dimethyl-sulfoxide). Amplifications were performed using the reaction profile: 95°C for 3 min, followed by 50 cycles at 95°C for 15 s and 60°C for 1 min in a 7900 HT sequence detector (Applied Biosystems, CA, USA) and were analyzed by a sequence detector system (SDS 2.4; Applied Biosystems).

### Statistical analysis

Patients with adequate samples for analysis were randomly divided into a Discovery cohort for separate global DNA methylation (*n* = 80) and gene-specific DNA methylation (*n* = 116) analyses and a Validation cohort for combined global and gene-specific DNA methylation analyses (Supplementary Figure 3). The revised Sydney criteria were used for gastritis classification (Lash, 2013 #166). Analyses were adjusted for *Helicobacter pylori* infection status, age and sex. Disease status was a binary variable indicating gastritis vs gastric adenocarcinoma based on pathological diagnosis. Subset analyses were performed in a subset of samples that were misdiagnosed by endoscopists. Global DNA methylation was measured on genomic DNA and analyzed as a continuous variable. Epigenome-wide and gene-specific DNA methylation was measured on bisulfite converted DNA and analyzed as continuous variables. Quantitative methylation specific PCR was used to measure promoter methylation of *IRF4*, *ELMO1*, and *MSC*.

The primary outcome indicator was a binary variable to identify gastric adenocarcinoma (GC) from gastritis cases. We also used the level of metaplasia as a proxy to identify gastritis patients with higher risk of developing GC. We conducted ordinary and logistic regression and analysis of variance, Fisher exact test and other tests of hypothesis to analyze the data. All data was analyzed and managed using STATA 13 (Statacorp, Texas, USA) and results with a *p* < 0.05 were considered as statistically significant. Student's *t*-test or ANOVA test were used for analyzing distributions or variances, respectively. Additional global and epigenome-wide analyses were performed in subset of patients from Peru (*n* = 201) for whom we also had a diagnosis provided by the endoscopists. In addition, epigenome-wide analyses were performed in 295 gastric cancer samples and 11 normal gastric epithelium samples from the Cancer Genome Atlas (TCGA) project.

## SUPPLEMENTARY FIGURES AND TABLES



## Abbreviations

GDMI: Global DNA Methylation Index
GC: Gastric cancer
NBI: Narrow-band imaging
LINEs: Long interspersed nuclear elements; *Helicobacter pylori*
