# Supplementary material for: Early detection of gastric cancer using global, genome-wide and IRF4, ELMO1, CLIP4 and MSC DNA methylation in endoscopic biopsies
Source: Oncotarget. 2017 Mar 16;8(24):38501–16. doi: 10.18632/oncotarget.16258 (PMC5503549; doi:10.18632/oncotarget.16258)
Supplement: Supplementary file 1 [file oncotarget-08-38501-s001.pdf]

Early detection of gastric cancer using global, genome-wide and *IRF4, ELMO1, CLIP4* and *MSC* DNA methylation in endoscopic biopsies

Supplementary Materials

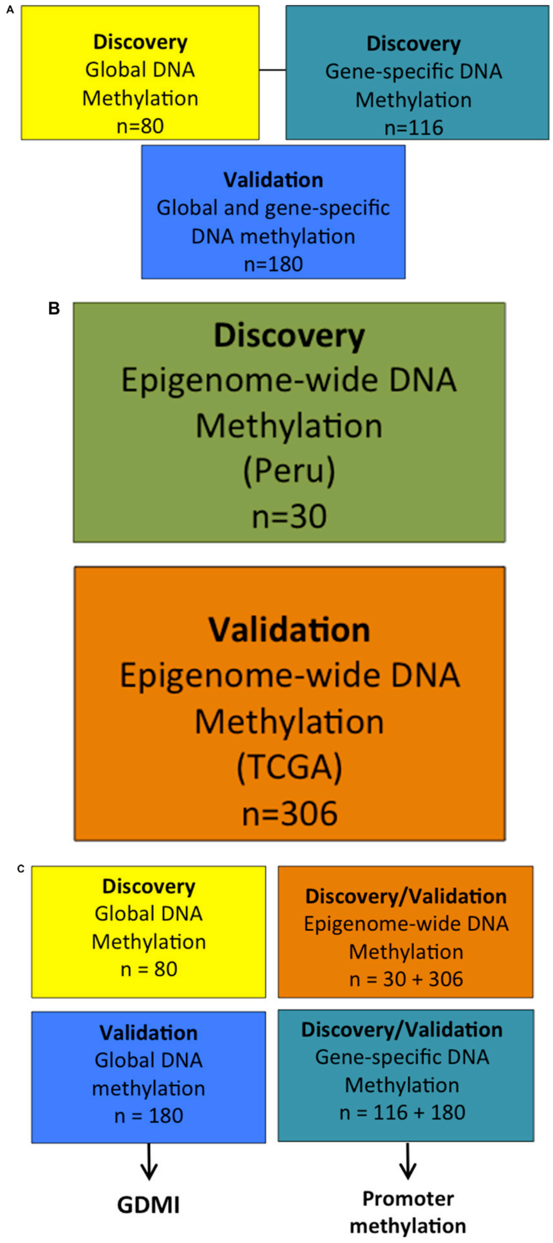

Supplementary Figure 1: (**A**) Discovery (Global n = 80; Gene-specific n = 116) and Validation (Global and Gene-specific methylation n = 180) data sets. (**B**) Discovery (Epigenome-wide n = 30 patients from Peru) and Validation (Epigenome-wide n = 306 patients from the Cancer Genome Atlas project) data sets. (**C**) Global DNA methylation analyses (Discovery (n = 80 and Validation n = 180) were performed to obtain the Global DNA Methylation Index cut-off levels for gastric cancer; Epigenome-wide DNA methylation analyses (Discovery n = 30 and Validation n = 306) were performed to identify differentially methylated promoters. These were used to develop quantitative Methylation Specific PCR (qMSP) primers and probes that were then tested in a Discovery cohort (n = 116) and a Validation cohort (n = 180).

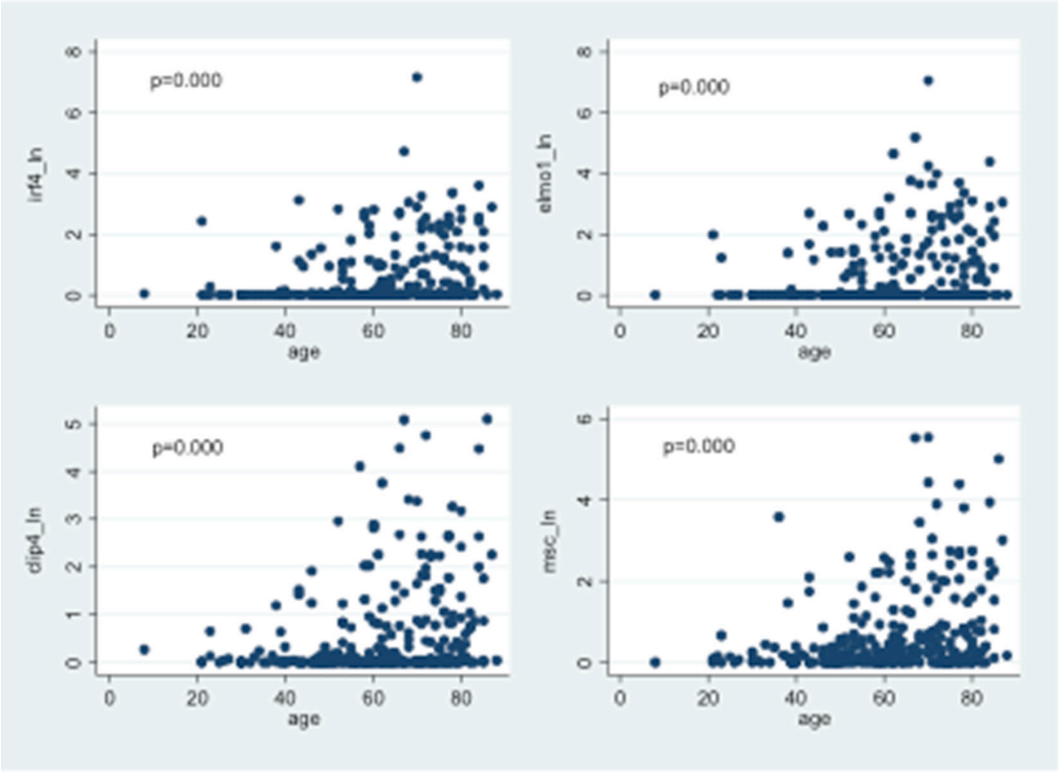

Supplementary Figure 2: Correlation between the natural logarithm of *IRF4, ELMO1, CLIP4* and *MSC* methylation and age.

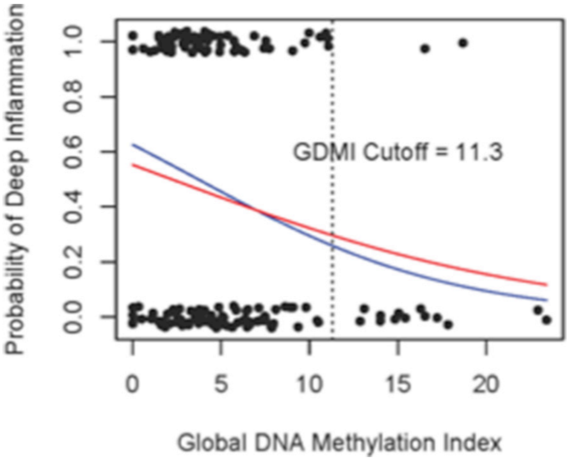

Supplementary Figure 3: The association between GDMI and depth of inflammation. The unadjusted probability of deep inflammation by GDMI is shown by the red line. The blue line is drawn after age adjustment.

Supplementary Table 1: Study participants characteristics

Variable	Obs	Mean (Std Dev)	Min	Max
sex	361	43% Male		
age	362	61.2 (14.9)	18	88
Case-control	376	34% Cases		
**Methylation:**				
Global	259	4.9 (4.04)	0	23.43
IRF4	296	6.47 (74.27)	0	1270.8
ELMO1	290	7.93 (69.43)	0	1157.2
CLIP4	294	3.9 (17.16)	0	162.67
MSC	295	4.88 (23.81)	0	254.46
**Sydney Criteria:**				
Intestinal metaplasia	241	19.09% > 0%, 12.45% > = 10%		
Glandular atrophy	234	12.8% > normal		
Inflammation depth	236	33.90% > superficial		
inflammation level	239	62.34% > normal		
activity	239	55.65% > normal		

Supplementary Table 2: Age and sex distribution by outcome variables

	% Male	Age	Obs
Gastritis	38.98	59.5	236
Gastric cancer	51.64	64.5	122
Metaplasia = 0 (gastritis patients)	37.63	58.1	186
Metaplasia > 0 (gastritis patients)	44.44	65.5	45

Supplementary Table 3: Global DNA methylation index (GDMI) as a tool to identify gastric cancer patients in endoscopic biopsies

	GDMI cutoff = 6.5		GDMI cutoff = 9	
	Cancer vs Gastritis	Metaplasia < 10 vs Metaplasia > = 10	Cancer vs Gastritis	Metaplasia < 10 vs Metaplasia > = 10
**sensitivity**	84.78%	87.50%	100%	96.3%
**Specificity**	32.82%	35.09%	11.70%	13.46%
**Pos. Pred. value**	30.71%	15.91%	29.36%	16.15%
**Neg. pred. value**	86.00%	95.24%	100%	95.46%

Supplementary Table 4: Correlation between various gene-specific methylation, age and sex

Log of methylation in:	IRF4	ELMO1	CLIP4	MSC	age	sex
IRF4	1					
**N**	295					
ELMO1	0.8948	1				
**N**	289	289				
CLIP4	0.6308	0.7192	1			
**N**	294	288	294			
MSC	0.7718	0.7829	0.7177	1		
**N**	295	289	294	295		
**age**	0.2365	0.2463	0.2679	0.2934	1	
**N**	282	276	281	282	362	
**sex**	0.0492	0.0001	0.0143	0.0032	0.0296	1
**N**	281	275	280	281	359	361

Supplementary Table 5: Summary statistics for global DNA methylation index in gastric cancer case control study in perú

	Cases	Controls
**Sample size**	50	151
**Range**	0.0–9.7	0.0–23.4
**Mean**	3.7	5.7
**Median**	3.4	4.2
**Standard Deviation**	2.5	4.5
**Interquartile range**	3.9	5.0

Supplementary Table 6: Classification of control study group according to pathological characteristics ($n = 151$)

	Superficial Inflammation	Deep Inflammation
**Level of Inflammation**		
Mild	41	18
Moderate	33	34
Severe	12	12
**Atrophy**		
Negative	85	40
Positive	2	24
**Intestinal Metaplasia**		
Negative	83	34
Positive	4	30
**Helicobacter Pylori**		
Negative	33	36
Positive	54	27

Supplementary Table 7: Primers ad probe sequences for *CLIP4, ELMO1, IRF4* and *MSC*

GENE	BISULFITE SEQUENCING	MSP	PROBES
CLIP4	F 5′-GCTGCCAGCAGGGAACTGGTGGAT-3′	F 5′-TTAAGACGGGTAAGATTAGGTTTTC-3′	5′-CGCCGCGAAACCAAACGACCCC-3′
	R 5′-CACTCCCCAACAATCAAATAATC-3′	R 5′-CCAACAATCAAATAATCGAACG-3′	
ELMO1	F 5-′AGGCAAAGTGCAGTGGGCAG-3′	F 5′-GACCAGGCAGGGTGCGGGCG-3′	5′-CGAACGCTACGAAATTACAACCG-3′
	R 5′-5CTTCCTCCTGCTGGCCAGAGAC-3′	R 5′-AAGGCATCCGGAGATCAGCCGCG-3′	
IRF4	F- 5′-TGTGAGGTTGATATTAGAGAGGATT-3′	F 5′-TTTAGGGTAGCGTAGGGTATTTC-3′	5′-CGAAACTCCAAAACGAAACGCGCTC-3′
	R 5′-AACCTTAAAACCCAAACCAAC-3′	R 5′-GAACAAAACCAAAAAAACGAA-3′	
MSC	F 5′-TGGAAAGACTGGCAGGCAAGCAGAG-3′	F 5′-ATTATTTGGGGGTAGAAGTATCGTC-3′	5′-CCGACGACCCTTCAACATACTAC-3′
	R 5′-ATCTGTACTCCTTGGAACAAACCAC-3′	R 5′-TACCGAAAAACGATTAATAAAAACG-3′	
